# Unveiling the potential of Urolithin A in Cancer Therapy: Mechanistic Insights to Future Perspectives of Nanomedicine

**DOI:** 10.7150/ntno.110966

**Published:** 2025-04-22

**Authors:** Vinita Karumuru, Anupam Dhasmana, Narsimha Mamidi, Subhash C. Chauhan, Murali M. Yallapu

**Affiliations:** 1 Micronutrient Information Center, Linus Pauling Institute, Oregon State University, Corvallis, OR 97331, USA; 2 Division of Cancer Immunology and Microbiology, Medicine and Oncology Integrated Service Unit, School of Medicine, University of Texas Rio Grande Valley, McAllen, TX 78504, USA; 3 South Texas Center of Excellence in Cancer Research, School of Medicine, University of Texas Rio Grande Valley, McAllen, TX 78504, USA; 4 Wisconsin Center for NanoBioSystems, School of Pharmacy, University of Wisconsin, Madison, WI, USA

**Keywords:** urolithin A, anticancer agent, chemoprevention, chemosensitizer, nanoparticles, adjuvant therapy

## Abstract

Cancer remains a leading global health challenge, with high mortality rates persisting despite significant advancements in therapeutic interventions. Major obstacles, including systemic toxicity, therapy resistance, metastasis, and relapses, emphasize the urgent need for safer, more effective, and readily accessible treatment strategies. Among emerging alternatives, natural bioactive compounds have gained considerable attention because of their diverse therapeutic potential and lower toxicity profiles. Urolithin A (UA), a metabolite derived from ellagic acid through gut microbiota metabolism, has emerged as a compelling anticancer agent. UA has multiple mechanisms of action, including the regulation of autophagy, enhancement of mitochondrial function, and inhibition of tumor progression and metastatic pathways. Additionally, its chemo-, immuno-, and radio-sensitization properties further increase its therapeutic advantages. Nanotechnology-driven approaches, such as nanoparticle formulations, lipids, and powder formulations, have successfully increased the solubility, stability, bioavailability, precise targeted delivery to cancer tissues, and overall therapeutic benefits of these materials. This review comprehensively explores the anticancer mechanisms of UA, its modulatory role, and advances in nanoformulation strategies, highlighting its potential as a next-generation therapeutic agent for improved cancer treatment and prevention.

## 1. Introduction

**Figure 1:**
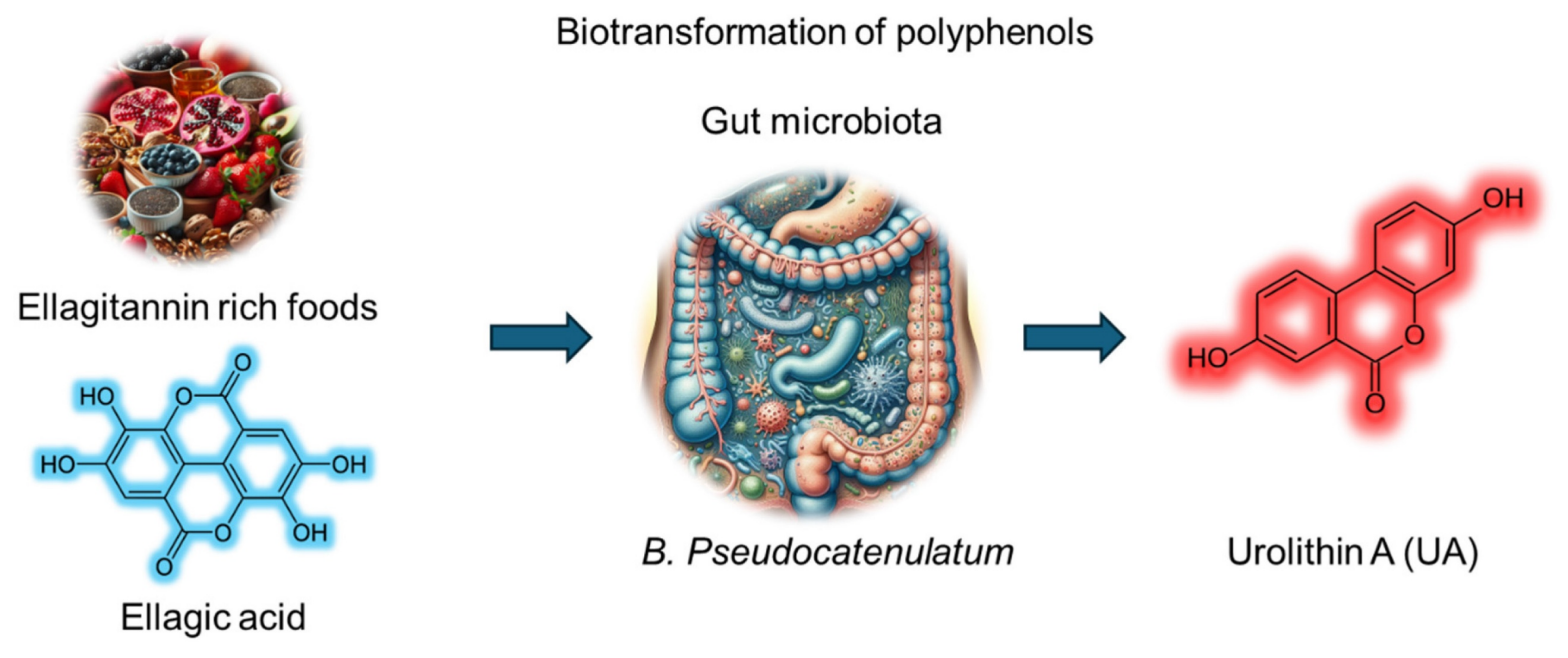
Schematic representation of the biotransformation of ellagitannin rich foods containing ellagic acid (polyphenols) into urolithin A *via* the gut microbiota utilizing *B. Pseudocatenulatum*.

Urolithin A (UA) is a metabolite derived from the biotransformation of polyphenols such as ellagitannins and ellagic acid by the gut microbiota. UA has emerged as a promising bioactive compound with diverse therapeutic potential. Dietary sources rich in UA precursors include fruits (pomegranates, raspberries, strawberries, and blackberries), walnuts and pecans, and plant-based products (certain tropical fruits, medicinal plants, and herbal teas) **(Figure 1).** UA, chemically identified as 3,8-dihydroxybenzo[c]chromen-6-one (molecular formula, C_13_H_8_O_4_ and molecular weight, 228.20 g/mol), belongs to the benzo[c]chromen-6-one chemical family. Its planar aromatic group includes hydroxyl groups at positions 3 and 8, contributing to its moderate hydrophilicity and antioxidant capacity. While sparingly soluble in water, UA shows better solubility in organic solvents, such as ethanol and dimethyl sulfoxide. It is stable under physiological conditions but prone to degradation at extreme pH values or high temperatures. The biotransformation of ellagitannins or ellagic acid to UA depends on specific gut microbial enzymes **(Figure 1)**. However, only 40% of individuals possess the requisite microbiota composition. Factors such as age, diet, and dysbiosis influence UA production, and its pharmacokinetics are highly variable across individuals. Plasma and tissue distribution studies suggest that UA achieves measurable concentrations, supporting its systemic availability and therapeutic applications. Despite its origin as a natural metabolite, UA has gained increasing attention for its pharmacological properties, including anti-oxidant, anti-inflammatory, and anti-cancer activities.

Given the multifaceted actions of UA, it is a promising candidate for therapeutic development across multiple medical fields. One of its primary mechanisms of action is mitophagy, a specialized form of autophagy that selectively targets and eliminates damaged mitochondria, thereby enhancing cellular bioenergetics and metabolic function 1-2. This inherent property contributes to the delay of aging processes and the mitigation of age-related functional decline. Consequently, UA has potential as a key component in antiaging interventions. Additionally, by improving mitochondrial function, UA can counteract age-related muscle wasting and enhance physical performance, making it a valuable therapeutic for improving muscle health and combating sarcopenia 3. The impact of UA on mitochondrial health and energy metabolism renders it a relevant molecule in the treatment of metabolic disorders (obesity, type 2 diabetes, and metabolic syndrome) 4. UA has neuroprotective properties because of its ability to reduce neuroinflammation, improve mitochondrial function, and mitigate oxidative stress, suggesting its potential application in neurodegenerative diseases (Alzheimer's disease, Parkinson's disease, and other age-related cognitive disorders) 5-7. In addition to these health benefits, emerging evidence highlights the ability of UA to reduce lipid peroxidation, combat oxidative stress, and improve endothelial function, promoting its role in cardiovascular health (aiding in the prevention of atherosclerosis, hypertension, and other cardiovascular diseases) 8-9. UA also exerts anti-inflammatory effects by suppressing the production of proinflammatory cytokines, such as TNF-α and IL-6, which can be employed for the management of chronic inflammatory conditions (such as rheumatoid arthritis and inflammatory bowel diseases) 10-11. Given that UA formation and bioactivity are influenced by the gut microbiota, its supplementation could promote a healthier gut microbiome, with potential therapeutic benefits for a wide range of conditions, including irritable bowel syndrome. Finally, UA has potent anticancer effects through cell cycle arrest, apoptosis induction, and the modulation of oncogenic signaling pathways. Its ability to regulate the tumor microenvironment by inhibiting angiogenesis and inflammation further underscores its potential as a therapeutic agent in cancer treatment.

**Figure 2:**
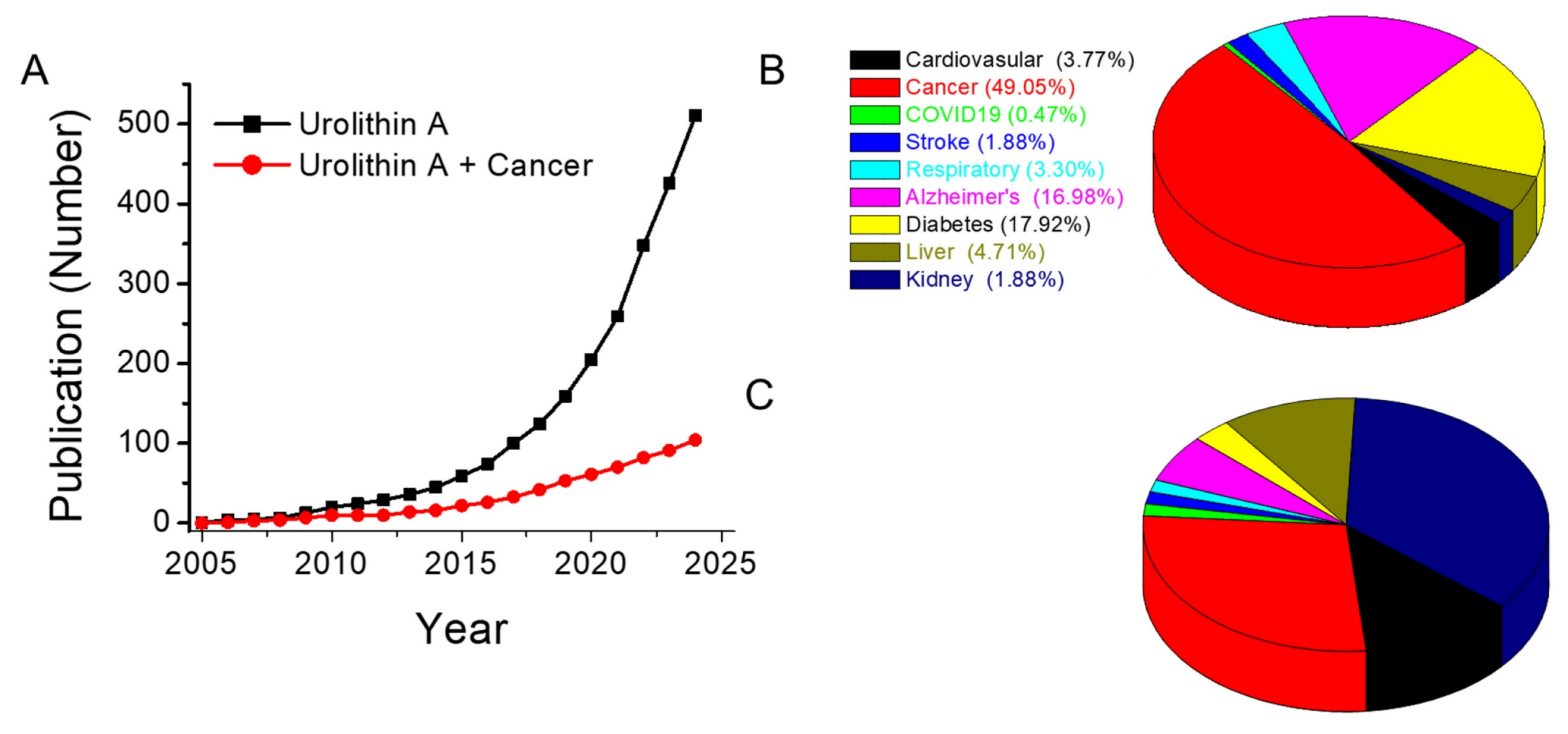
Scientific implications of urolithin A in biomedical research, other diseases, and cancer research. **A.** Line graph illustrating the trend in publications related to UA across various diseases (black line) and specifically in cancer research (red line).** B.** Pie chart depicting the contribution of UA research across the top ten diseases in the United States, apart from accidental injuries, for which no UA-related publications were identified.** C.** Pie charts showing the distribution of studies on UA involvement in specific cancer types. Data were obtained from PubMed (National Center for Biotechnology Information, U.S. National Library of Medicine) via the search terms “Urolithin A” and “specific disease” (B) or “specific cancer” (C). The search was conducted on November 22, 2024, and included studies published from 2005--2024, with no specific exclusion criteria applied.

The extensive therapeutic potential of UA across various medical applications underscores the necessity for further clinical research and development to fully harness its benefits. A systematic review was conducted following the Preferred Reporting Items for Systematic reviews and Meta-Analyses guidelines. This comprehensive search was performed on PubMed via keywords such as "urolithin A", which resulted in the identification of 512 articles focused on all biomedical research applications of UA **(Figure 2A).**

These findings highlight that UA has been the subject of active research since 2005, with a substantial body of work exploring its biological and therapeutic properties **(Figure 2A).** An analysis of the top ten medical applications of UA revealed its involvement in diverse therapeutic areas, with the highest concentration of studies in cancer (104), followed by diabetes (38), Alzheimer's disease (36), liver (10), respiratory (7), kidney (4), stroke (4), and infectious (COVID-19, 1) diseases **(Figure 2B).** Notably, over 49% of the research on UA has focused on its potential as a cancer therapeutic, confirming its prominence as a promising candidate in oncology. A more detailed breakdown of these studies revealed that UA has been predominantly studied in the context of breast, colorectal, glioma, pancreatic, and prostate cancers **(Figure 2C).** However, there is a noticeable gap in the exploration of the effects of UA in other cancer types.

UA has gained significant attention because of its promising therapeutic potential in the treatment and prevention of cancer. Despite its increasing recognition within the field of cancer therapeutics, a comprehensive review of its extensive applications in oncology is lacking **(Table 1).** This review consolidates a broad spectrum of original research studies, systematic reviews, and meta-analyses, focusing on both preclinical and clinical investigations evaluating the anticancer potential of UA. The reviewed studies encompass cellular and animal models, as well as human studies, with a particular emphasis on cancer-related research. These studies highlight various mechanisms by which UA exerts its anticancer effects, including enhancing mitochondrial function, inducing apoptosis, and inhibiting tumor growth. Additionally, this review incorporates research on nanoparticle-based delivery systems, which play a critical role in optimizing the therapeutic efficacy of UA and ensuring targeted drug delivery to tumor sites.

A systematic analysis of the literature reveals that over 49% of UA-related research is dedicated to oncology, emphasizing its potential as a promising anticancer agent. The innovation of this review article is primarily integrating findings of preclinical and clinical studies, elucidating the mechanistic actions of UA, including mitochondrial function enhancement, apoptosis induction, and tumor growth inhibition. While UA has been extensively investigated in breast, colorectal, glioma, pancreatic, and prostate cancers, its therapeutic translation remains a challenge due to limitations in delivery and bioavailability. The key novelty of this review lies in its comprehensive assessment of UA within the framework of nanomedicine, with a particular focus on its oncological applications. This work underscores the critical role of nanotechnology-driven drug delivery systems in improving UA's pharmacokinetic properties, stability, and tumor-specific targeting. By addressing these challenges, this review aims to facilitate the advancement of UA-based nanomedicine strategies for enhanced cancer therapeutics.

## 2. Role of urolithin A in cancer

**Figure 3:**
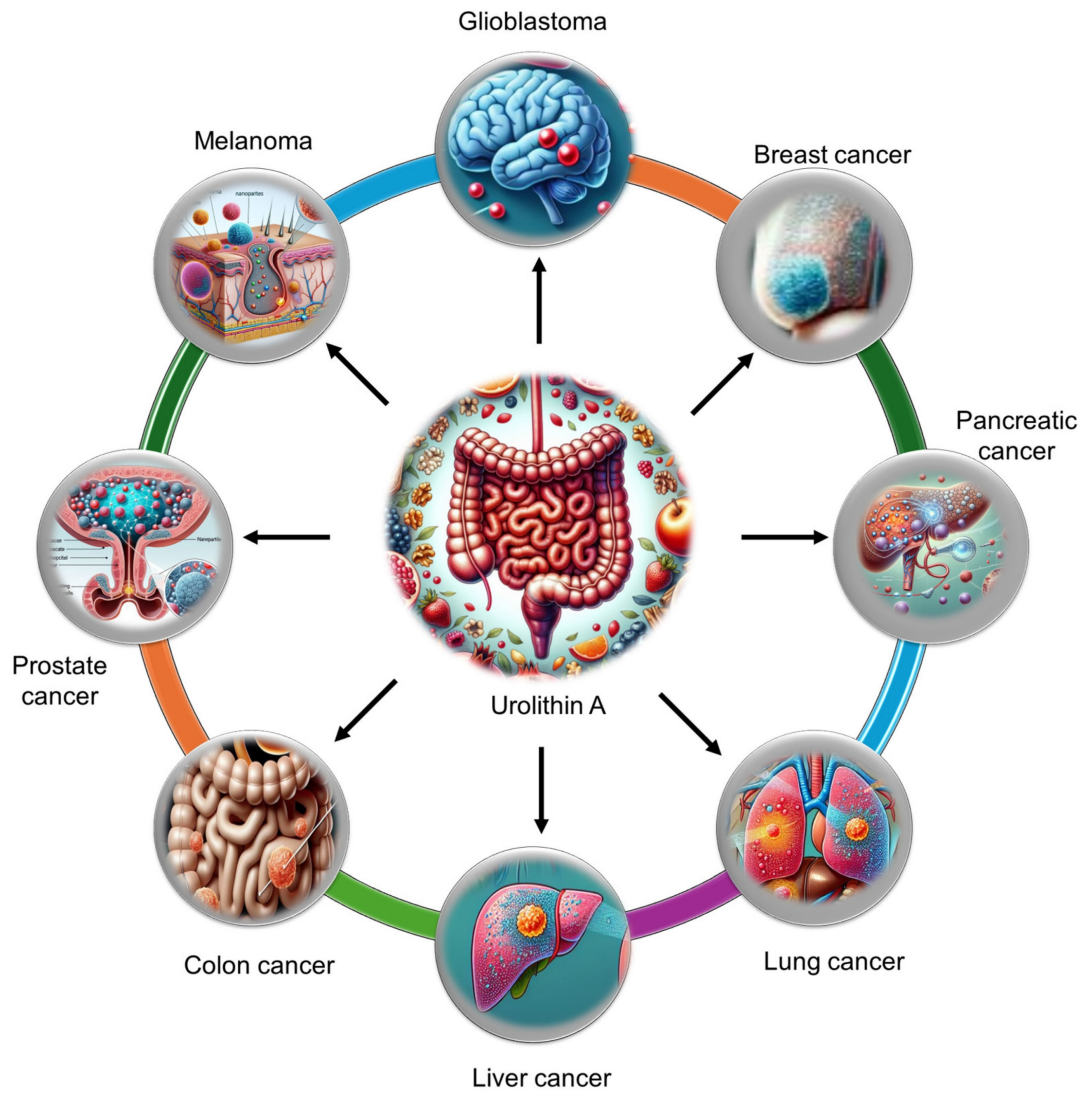
Urolithin A can efficiently reach specific tumor tissues and aid in the treatment of various tumor types, including gliomas, melanomas, and breast, liver, lung, pancreatic, colon, and prostate tumors.

Extensive research has demonstrated the potent anticancer and chemopreventive properties of UA across a wide spectrum of malignancies. UA has been investigated in various cancers, including breast, colorectal, hepatic, pulmonary, pancreatic, prostate, glioblastoma, and bladder cancers **(Figure 3).** The ability of UA to modulate key oncogenic pathways has been revealed. The antitumor activity of UA is mediated through multiple mechanisms, such as the induction of cell cycle arrest, the enhancement of mitophagy and autophagy, the inhibition of matrix metalloproteinases (MMPs), and the promotion of apoptosis. Additionally, UA influences critical signaling pathways, including the PI3K/AKT/mTOR, p53/TIGAR, Wnt, and FOXO1/FOXO3-FOXM1 pathways, contributing to its anticancer effects.

UA restricts tumor progression by reducing cancer cell proliferation, migration, and invasion. Its role as a selective estrogen receptor modulator (SERM) further contributes to its tumor-suppressive effects, especially in hormone-dependent cancers. Furthermore, UA modulates the inflammatory tumor microenvironment by downregulating the expression of proinflammatory cytokines, such as IL-6 and TNF-α, which play crucial roles in tumor growth and metastasis. UA also impedes mechanisms of drug resistance, particularly through the inhibition of ABCG2-mediated drug efflux, thereby increasing the effectiveness of chemotherapy agents.

**Figure 4:**
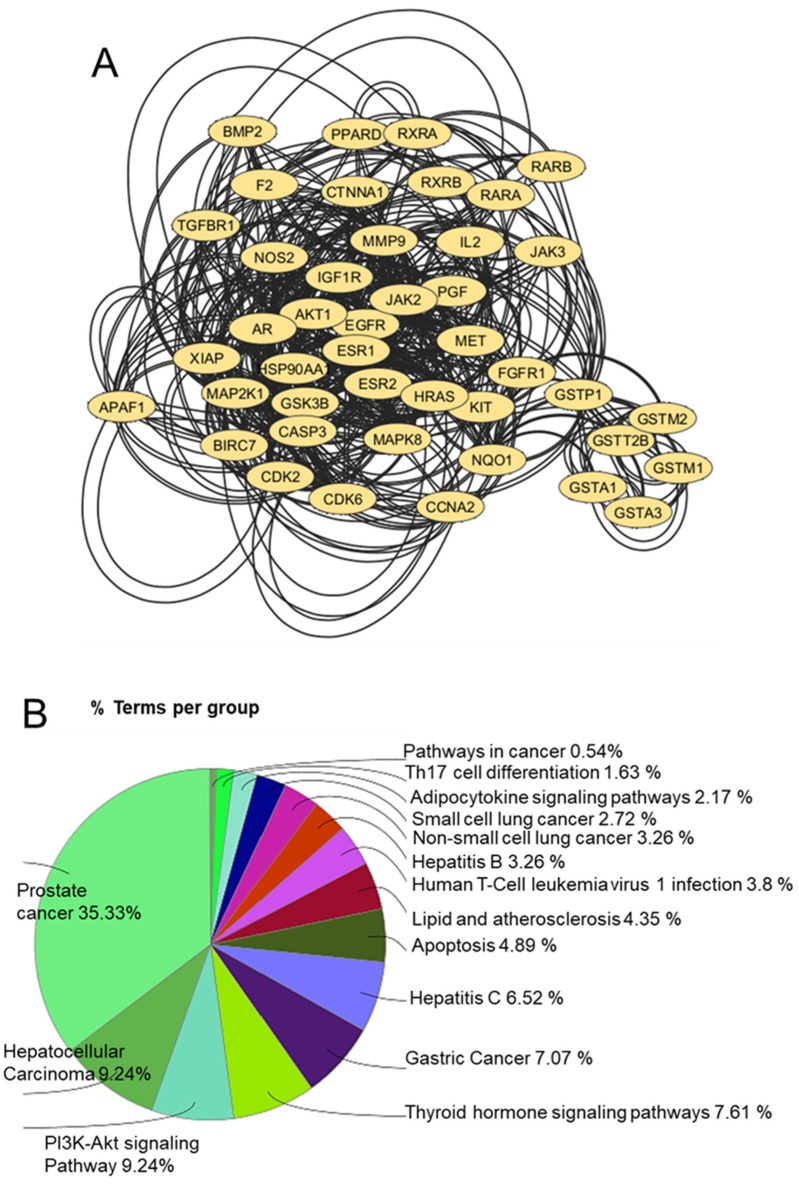
**A)** Prefuse force-directed layout of 43 proteins that were submodulated and enriched for cancer pathways. **B)** Functional enrichment of all 43 proteins in various subtypes of cancer and their related pathways.

Docking tools play an integral role in elucidating the biological activity of UA by identifying its potential protein targets and mapping its pharmacological landscape. The reverse docking approach is particularly useful for pinpointing interactions between UA and human proteins, providing a foundation for understanding its therapeutic potential and designing subsequent experiments. By employing the 3D format of UA in the Tripos Mol2 format, a comprehensive global reverse docking search across human proteins enabled the identification of approximately 246 interaction targets, revealing the diverse protein-binding capabilities of UA 22. Pharmacophore modeling, a critical 3D feature of the chemical structure of UA, is instrumental in defining its probable targets. According to the IUPAC, a pharmacophore represents the spatial and electronic properties required for effective interactions with specific biological targets 23. This modeling approach revealed the optimal protein matches for UA, guiding the docking process toward identifying biologically relevant interactions. STRING database analysis further enriched the understanding of the impact of UA by highlighting interprotein connections among the 246 identified targets. Functional enrichment and network submodulation refined the focus to 43 proteins implicated in cancer pathways, namely, AKT1, APAF1, AR, BIRC7, BMP2, CASP3, CCNA2, CDK2, CDK6, CTNNA1, EGFR, ESR1, ESR2, F2, FGFR1, GSK3B, GSTA1, GSTA3, GSTM1, GSTM2, GSTP1, GSTT2B, HRAS, HSP90AA1, IGF1R, IL2, JAK2, JAK3, KIT, MAP2K1, MAPK8, MET, MMP9, NOS2, NQO1, PGF, PPARD, RARA, RARB, RXRA, RXRB, TGFBR1 and XIAP. Among these proteins, AKT1 has emerged as the most centrally connected protein, emphasizing its pivotal role in the anticancer potential of UA. Tools such as Cytoscape and ClueGO facilitated the visualization and functional analysis of these networks, revealing the involvement of UA in pathways related to prostate cancer, hepatocellular carcinoma, lung cancer, and the PI3K-AKT signaling pathway **(Figure 4).**

In silico computational docking studies further confirmed the binding affinity of UA for various cancer-related molecular targets, confirming its potential as a promising chemotherapeutic agent. Through these docking tools and bioinformatics platforms, researchers have gained critical insights into the interaction landscape of UA, highlighting its potential as a therapeutic agent, particularly in anticancer applications. This integrative approach underscores the power of computational tools in modern drug discovery and pharmacology research. In addition, UA has been shown to improve therapeutic outcomes in drug-resistant cancers and enhance the efficacy of both chemotherapy and immune checkpoint inhibitors. These findings collectively underscore the therapeutic potential of UA in cancer treatment, positioning it as a valuable candidate for further clinical exploration.

### 2.1. Breast cancer

Breast cancer remains one of the most prevalent and challenging malignancies worldwide, necessitating the development of novel therapeutic strategies. This section aims to discuss the mechanisms of action of UA, its effects on cellular pathways, and its potential to enhance breast cancer therapy. UA exerts significant antitumor activity by modulating critical molecular pathways in breast cancer. It inhibits cell proliferation, induces apoptosis, and promotes the maintenance of cellular homeostasis by regulating key signaling cascades, including the mTOR, PI3K/AKT, and nuclear factor kappa B (NF-κB) pathways 24-26. In addition, UA enhances autophagy through mitophagy by promoting the nuclear translocation of transcription factor EB (TFEB), functions as a SERM, and reduces inflammatory responses 24, 27. These multifaceted roles highlight their potential for breast cancer management.

Preliminary investigations revealed that urolithins are metabolites derived from the intake of pomegranate ellagitannins that exhibit aromatase-inhibitory activity 28. In addition, urolithins also inhibit testosterone-induced breast cancer cell proliferation. A follow-up investigation revealed that UA exhibits both estrogenic and antiestrogenic activities, indicating that it is a potential phytoestrogen 29. UA inhibited key signaling pathways, such as the NF-κB and PI3K/AKT pathways, which are critical for tumor progression. In cell culture studies, UA reduced the expression of inflammatory markers by 50-60%, enhancing its chemopreventive efficacy. UA also promoted apoptosis via the activation of caspases and the downregulation of survival proteins such as Survivin. In a subsequent study, it was identified as a substrate of the breast cancer resistance protein (BCRP/ABCG2), an essential efflux transporter associated with drug resistance in breast cancer 30. An experiment utilizing BCRP-transduced cells and selective inhibitors demonstrated that UA inhibited mitoxantrone transport in a dose-dependent manner, suggesting its potential to modulate ABCG2-mediated drug efflux and enhance the efficacy of chemotherapeutic agents.

In estrogen receptor-positive breast cancer, UA has a specific capacity to modulate estrogen receptor activity and reduce the oncogenic effects of 27-hydroxycholesterol 27. UA efficiently suppressed 27-HC-driven estrogen receptor activation by downregulating estrogen-responsive genes and estrogen response element activity. UA inhibited MCF-7 cell proliferation in vitro and significantly reduced 27-HC-induced tumor growth in vivo. It modulates the mitochondrial membrane potential to induce apoptosis. These findings establish UA as a dual-action SERM and therapeutic agent for ER-positive breast cancer.

A recent study highlighted the role of UA as an autophagy enhancer and a modulator of the breast cancer tumor microenvironment 24. UA promoted the TFEB by inhibiting its ubiquitination via mTOR-dependent pathways, thereby facilitating mitophagy and reducing mitochondrial stress. In breast cancer organoid models and tumor-associated macrophage cocultures, UA suppressed proinflammatory cytokines, including IL-6 and TNF-α, creating a tumor microenvironment with reduced inflammation. Molecular docking studies further revealed that UA interacts with the 14-3-3 protein, preventing its binding to TFEB and enhancing cellular homeostasis.

Another study demonstrated the dose-dependent cytotoxic effects of UA on hormone receptor-positive and triple-negative breast cancer cell lines, with significant inhibition of cell proliferation observed at concentrations as low as 100 µM 25. Mechanistic analyses revealed that UA induced cell cycle arrest by downregulating cyclin D1 and c-MYC and promoted apoptosis by increasing the expression of proapoptotic proteins such as Bax while reducing antiapoptotic BCL2 levels.

Molecular docking and enzyme inhibitory assays further confirmed that UA has anti-breast cancer potential 26. UA strongly interacts with cancer-associated enzymes such as α-amylase (-5.169 kcal/mol), α-glucosidase (-3.657 kcal/mol), and aldose reductase (-7.635 kcal/mol), reducing their activity through hydrogen bonding. Cytotoxicity studies revealed that the IC50 values of UA ranged from 392 to 566 µM across various breast cancer cell lines (SkBr3: 400 µM, MDA-MB-231: 443 µM, MCF-7: 392 µM, Hs578T: 418 µM, Evsa-T: 397 µM, BT-549: 530 µM, AU565: 566 µM, and 600 MPE: 551 µM), underscoring its broad-spectrum anticancer efficacy.

The physiological relevance of UA and its conjugates was confirmed through metabolic profiling of malignant mammary tissues 31. This study demonstrated that urolithins exert more substantial anti-proliferative effects than other polyphenols, such as hesperetin, resveratrol, dihydroresveratrol, and their conjugates do. While UA-associated metabolites were detected in mammary tissues following supplementation, no direct estrogenic or antiestrogenic activities were observed in specific breast cancer models 32.

### 2.2. Colon cancer

UA has gained significant attention as a promising chemical and chemopreventive agent for treating colorectal cancer (CRC). Owing to its diverse molecular mechanisms, UA influences critical cellular processes and signaling pathways associated with tumor progression and metastasis. A comprehensive analysis highlighting the anti-CRC potential of UA, supported by both in vitro and in vivo findings, as well as clinical evidence, is presented below.

**Figure 5:**
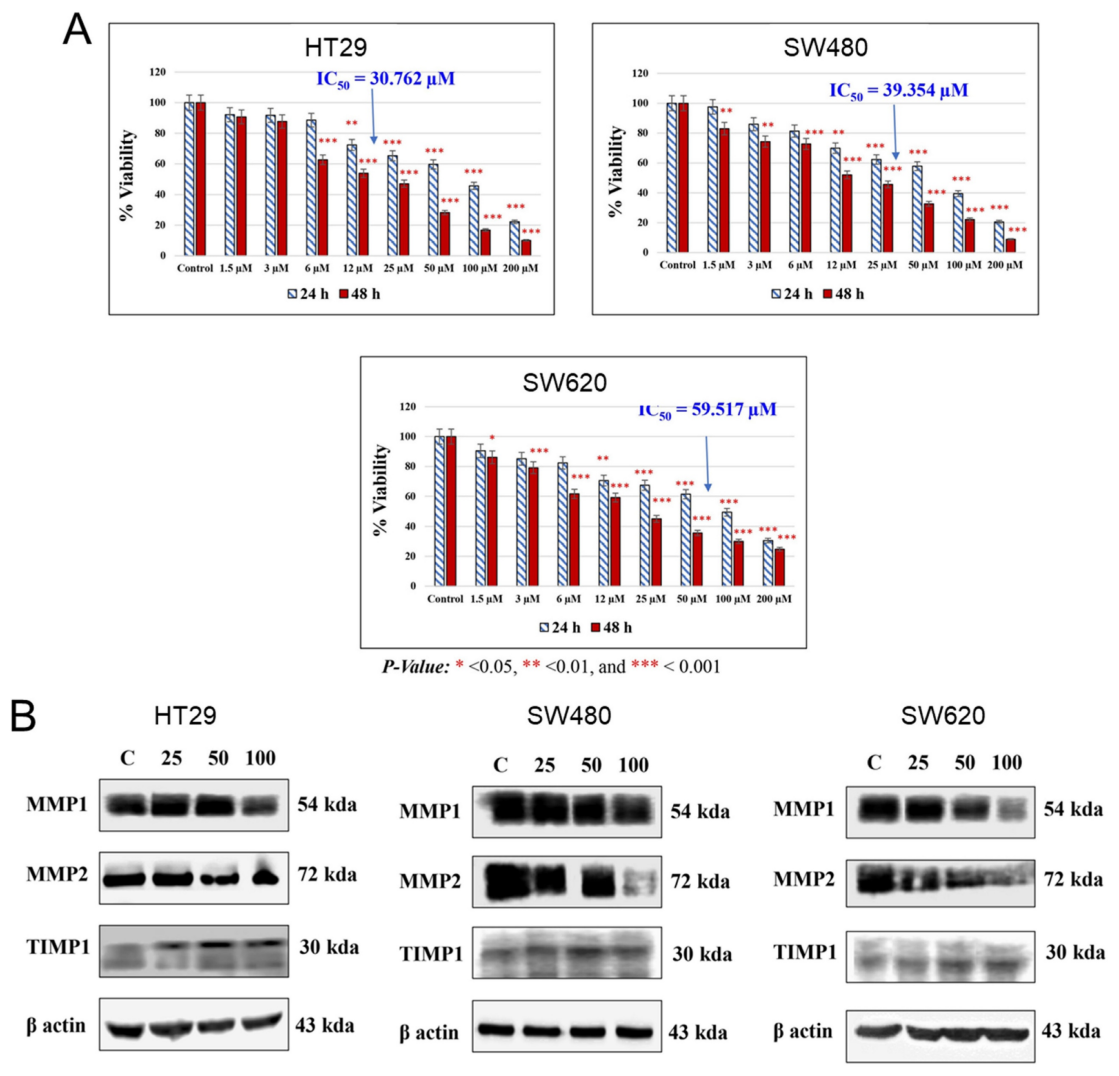
Cytotoxic and proteomic effects of UA on colorectal cancer cells. **A).** The AlamarBlue assay was conducted to assess UA-induced cytotoxicity in HT29, SW480, and SW620 cell lines at 24 and 48 hours. IC50 values were determined, with statistical significance set at *p* < 0.05 (**p** < 0.001 marked as ***). **B).** An immunoblot analysis evaluated the effect of UA (0, 25, 50, 100 µM) on metalloproteinase expression. Reproduced with permission from reference 35. Copyright 2024 John Wiley and Sons.

In 2009, the chemopreventive properties of UA were demonstrated in a Caco-2 cell line through its influence on gene expression and cellular functions 33. UA treatment led to decreased cell viability and induced cell cycle arrest at the S and G2/M phases, although it did not significantly promote apoptosis. Additionally, this compound modulates key MAPK signaling genes, such as FGFR2, EGFR, K-RAS, and c-MYC, and tumor suppressors, such as DUSP6 and Fos, as well as the cell cycle regulators CCNB1 and CCNB1IP1. Notably, UA effectively inhibits the Wnt signaling pathway, which is aberrantly activated in 90% of CRC cases 34. This activity was confirmed via a luciferase reporter assay in a human 293T cell line. The percent Wnt-activated response of UA (9 µg/ml) was superior to that of ellagitannin (28 µg/ml) and ellagic acid (19 µg/ml). Further investigations by El-Wetidy et al. 35 emphasized its role in suppressing the proliferation **(Figure 5A),** migration, metastasis, and clonogenic efficiency of HT29, SW480, and SW620 cells. UA downregulated the expression of matrix metalloproteinases (MMP1 and MMP2), which are critical for extracellular matrix degradation and metastasis, while the expression of their inhibitor tissue inhibitor of metalloproteinases 1 is increased **(Figure 5B).** These findings underscore the potential of UA to inhibit CRC metastasis by disrupting cellular invasion and colony formation.

UA has also been shown to inhibit 2,3,7,8-tetrachlorodibenzo-p-dioxin induced CYP1-mediated 7-ethoxy-resorufin-O-deethylase (EROD) activity and to suppress HT-29 cell proliferation in a dose- and time-dependent manner 36. This activity involves cell cycle arrest at the G0/G1 and G2/M phases and the induction of apoptosis. Another study confirmed that UA was capable of increasing CYP1A1 expression 2.9-fold in an EROD assay in Caco-2 cells 37. This activity was further supported by CYP1A1 mRNA expression in situ and in vivo in rat colon mucosa models. Cho et al. 38 further demonstrated that UA has anticancer effects on HT-29 cells by inducing cell cycle arrest (G2/M phase), increasing p21, and triggering apoptosis via the activation of caspases 8, 9, and 3, along with PARP cleavage.

Importantly, a recent study revealed that UA induces similar proliferation, cell cycle arrest, and apoptosis in Caco-2 cells, but these effects are considerably limited in CCD18-Co normal cells 39. A mechanistic study of Pirc rats with adenomatous polyposis coli mutations revealed a significant reduction in mucin-depleted foci and increased apoptosis in response to treatment with pomegranate mesocarp decoction 40. UA, a metabolite of pomegranate mesocarp, synergistically reduced COX-2 expression by ~70% and increased cleaved caspase-3 levels (~10-fold in cell cultures, +74% in adenomas, and +69% in normal mucosa), indicating that the proapoptotic and anti-inflammatory properties of UA promote tumor progression in high-risk CRC patients. Recent studies have further elucidated the molecular effects of UA, which induce reduced glycolytic activities via the p53/TIGAR axis 41, p53-dependent cellular senescence 42, and regulation of the FOXO3-FOXM1 axis 43, all of which contribute to its anticancer efficacy.

A clinical trial involving 52 CRC patients who consumed 900 mg of pomegranate extract per day for 15 days provided critical insights into UA bioavailability and activity 44. This analysis revealed higher metabolite concentrations in malignant tissue (up to 1671 ± 367 ng/g) than in normal colon tissue. These findings support the potential for UA delivery to colon tissues to achieve its anti-CRC activity.

### 2.3. Liver cancer

**Figure 6:**
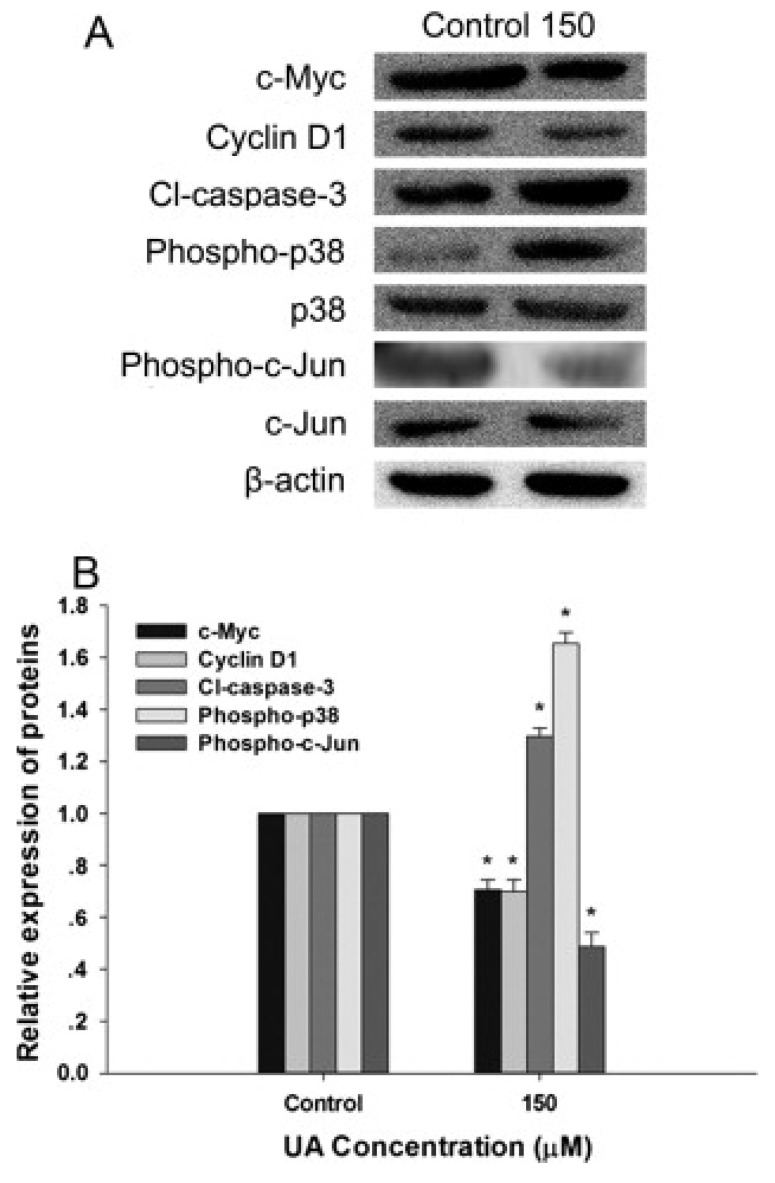
Urolithin A modulates β-Catenin/TCF pathway and apoptotic markers in hepatocellular carcinoma cell lines. An immunoblot analysis was performed to evaluate the effect of UA (150 μM) on β-catenin/TCF target proteins (c-Myc, Cyclin D1), apoptotic marker cleaved caspase-3, and p38-MAPK signaling after 24 h of treatment. **A)** Representative Western blot images and **B)** Quantification of protein expression levels normalized to β-actin. Data are presented as mean ± SD (p < 0.05, n = 3). Reproduced with permission from reference 45. Copyright 2015 Elsevier.

Hepatocellular carcinoma (HCC), the most common primary liver cancer, remains a significant global health challenge. UA has emerged as a promising candidate for the prevention and treatment of HCC, offering potent antiproliferative, antioxidant, and anti-inflammatory properties. UA has demonstrated potent antiproliferative and antioxidant effects on HepG2 human hepatoma cells 45. UA effectively reduces cell viability in a dose- and time-dependent manner by downregulating β-catenin signaling, along with its downstream mediators, c-MYC and Cyclin D1, while suppressing TCF/LEF transcriptional activation **(Figure 6)**. Concurrently, UA induces the expression of tumor suppressor proteins such as p53 and p38-MAPK and promotes apoptotic pathways via caspase-3 activation **(Figure 6)**.

In addition to its antiproliferative effects, UA demonstrates significant antioxidant activity by reducing reactive oxygen species levels and enhancing the activities of key antioxidant enzymes, such as superoxide dismutase and glutathione peroxidase. UA mitigates alcohol-induced metabolic dysfunction and hepatic ER stress via the modulation of major urinary protein 1 46. These actions mitigate oxidative stress, a critical factor in liver carcinogenesis, thereby protecting hepatic cells from further damage. The potential of UA extends beyond direct antitumor activity. It has been shown to ameliorate alcohol-induced metabolic dysfunction and hepatic endoplasmic reticulum (ER) stress via the modulation of major urinary protein 1. Furthermore, UA beneficially alters the gut microbiota composition, increasing populations of beneficial bacteria such as *Bacteroides sartorii, Parabacteroides distasonis,* and *Akkermansia muciniphila.* This microbiome-modulating capacity offers a novel therapeutic avenue for addressing alcohol-related liver diseases, which contribute to approximately 20% of liver cancer-associated fatalities.

### 2.4. Glioblastoma

**Figure 7:**
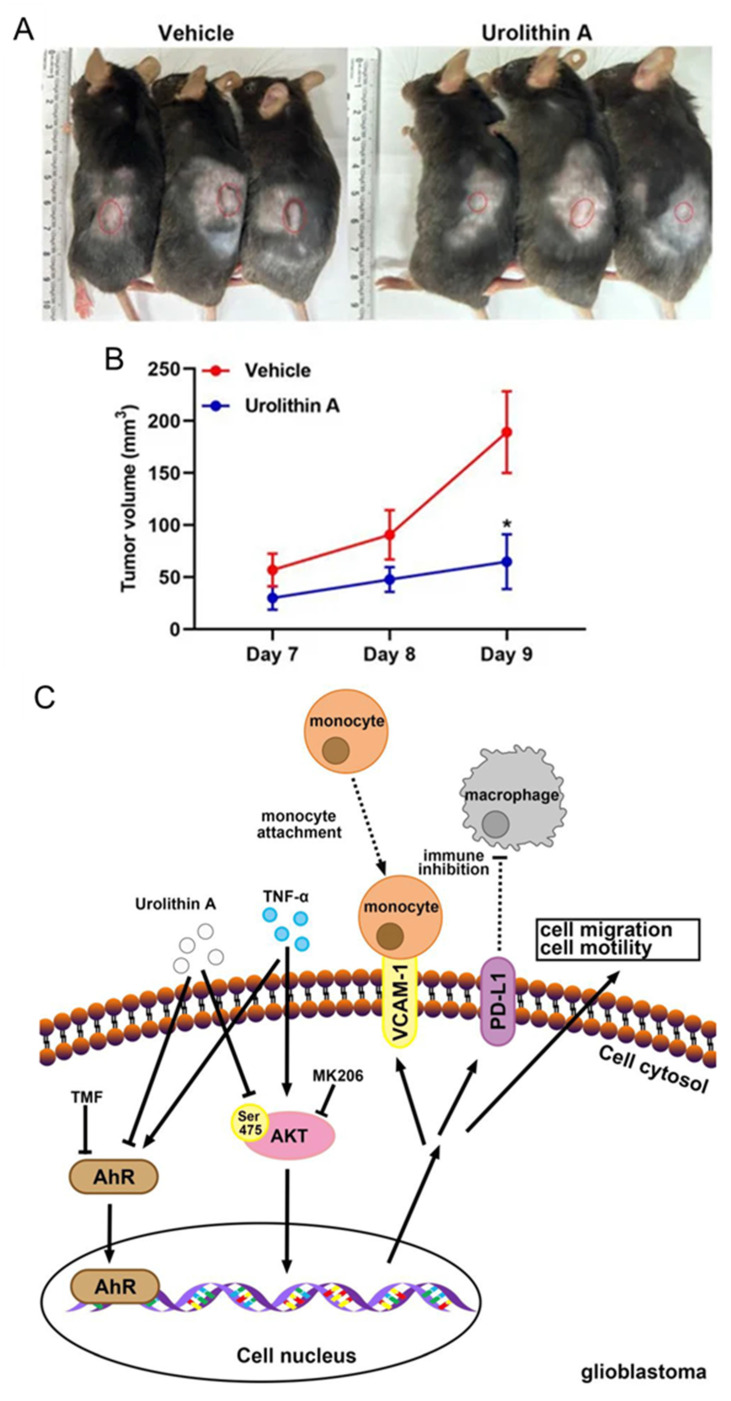
Urolithin A inhibits GBM growth and modulates tumor signaling pathways. **A)** ALTS1C1 GBM cells (1 × 10⁷) were subcutaneously injected into mouse flanks, followed by daily intraperitoneal administration of UA (40 mg/kg) or vehicle control. **B)** Tumor volume was measured daily, with data expressed as mean ± SEM (*p* < 0.05, Student's *t*-test). **C)** Schematic illustration of UA's mechanism in suppressing GBM progression by inhibiting TNF-α-induced VCAM-1 and PD-L1 expression while modulating Akt signaling to prevent tumor migration and growth. Reprinted with permission under the terms of the Creative Commons CC BY license from reference 48. Copyright © 2023, The Author(s), published by MDPI, Basel, Switzerland.

A comprehensive study evaluated the pharmacological and biological activity of UA in glioblastoma (GBM) using both an in vitro glioblastoma cell line and an in vivo xenograft mouse model 47. These results suggest that UA exerts significant anticancer activity by inhibiting glioblastoma cell proliferation, migration, invasion, and colony formation in U251 and U118 MG cells in a dose- and time-dependent manner. Mechanistically, UA induced cell cycle arrest and apoptosis while enhancing the expression of key tumor suppressors, including Sirtuin 1 (Sirt1) and Forkhead box protein O1 (FOXO1), both of which are crucial regulators of cancer cell dynamics. These effects are mediated through the modulation of the ERK and AKT signaling pathways, underscoring the impact of UA on critical molecular mechanisms in glioblastoma progression. Additionally, UA effectively suppresses glioblastoma cell migration, and epithelial mesenchymal transition (EMT) is closely associated with tumor aggressiveness 48 **(Figure 7)**.

UA also reduced TNF-α-induced expression of vascular cell adhesion molecule-1 and programmed death-ligand 1 in U251 and ALTS1C1 GBM cells. These regulatory effects are linked to the inhibition of the Akt and EGFR signaling pathways, as well as the modulation of the aryl hydrocarbon receptor signaling axis, which is implicated in glioblastoma malignancy **(Figure 7)**. In vivo studies further supported these findings, showing that UA administration significantly decreased tumor growth and weight in glioblastoma xenograft models **(Figure 7A-B)**. Notably, UA upregulated Sirt1 and FOXO1 expressions in tumor tissues, reinforcing its role in modulating pathways critical to tumor suppression **(Figure 7C)**. Moreover, the ability of UA to modulate inflammatory and immune-related pathways suggests a multifaceted approach for mitigating glioblastoma progression.

### 2.5. Pancreatic cancer

Pancreatic cancer, particularly pancreatic ductal adenocarcinoma (PDAC), remains one of the most aggressive malignancies with limited therapeutic options. Initial investigations revealed the ability of UA to affect the viability of pancreatic cancer cells treated with high micromolar concentrations, with evidence supporting its broader application in pancreatic cancer management 13. A study by Kucukkaraduman et al. 49 evaluated the antiproliferative effects of UA alongside seven other natural compounds across multiple cancer cell lines, including pancreatic, colon, and breast cancer cell lines. These findings suggest that UA induces mesenchymal-to-epithelial transition, a critical process in reducing tumor invasiveness. Seeram and Heber also reported that urolithins, including UA, can be useful in the treatment and prevention of neoplastic diseases 50. This patent expresses pancreatic cancer cell lines and pancreatic cancer-based xenograft mouse model studies.

Further studies by Nagathihalli et al. 17, 51 revealed UA as a novel naturally occurring molecule that targets major pathways in PDAC. UA displayed variable sensitivity in nine PDAC cell lines, with Capan-1 and HPAC being the most responsive (IC50 ~10 μM). It inhibits the PI3K/AKT/mTOR pathway, suppresses G1-phase cell cycle progression, and induces apoptosis without harming normal pancreatic cells. UA can reduce the levels of critical regulators of proliferation and survival (phosphorylation of AKT:T308 and p70S6K:T421/S424) in a dose-dependent manner. In vivo studies involving MiaPaCa2 and PANC1 xenografts revealed that UA treatment significantly inhibited tumor growth (MiaPaCa2: ~ 200 mm^3^ vs. control ~800 mm^3^ and PANC1: ~ 200 mm^3^ vs. control ~850 mm^3^), reduced proliferation, and induced apoptosis (cleaved Caspase-3) without toxicity.

**Figure 8:**
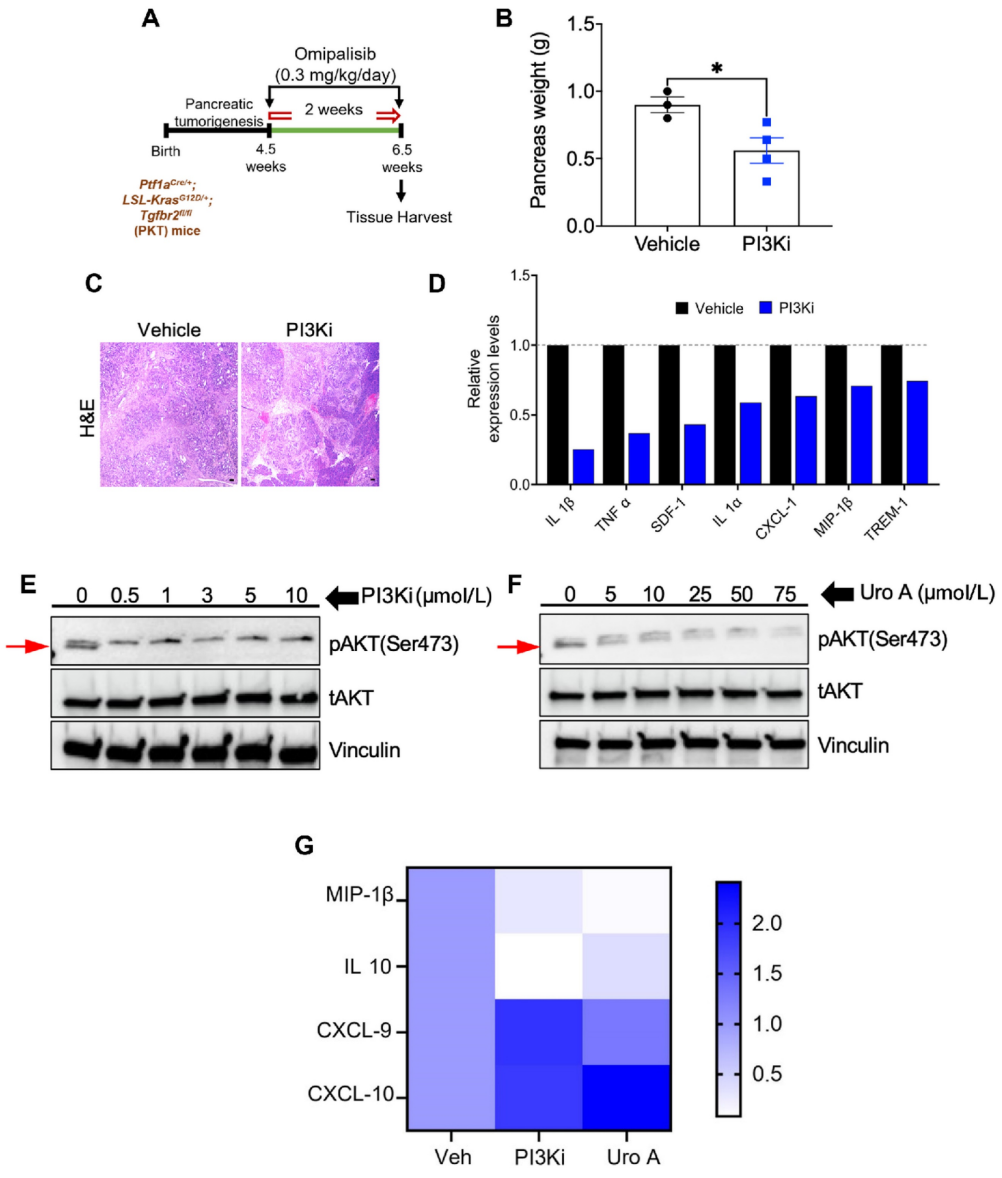
PI3K inhibition mimics UA's antitumor and immunomodulatory effects in PDAC. **A)** Diagram of PI3K inhibition using Omipalisib treatment in mice model. **B)** Graphical representation of pancreas weight after treatment (n = 3-4/group). **C)** H&E-stained tumor sections from both treatment groups. **D)** Cytokine profiling evaluation of pancreatic tumors post-treatment. **E-F)** Immunoblot analysis of pAKT and tAKT levels in murine KPC PDAC cell lines upon treatment with PI3Ki (0-10 μM) or UA (0-75 μM) for 3 hours. **G)** Heatmap illustrates fold changes in immunomodulatory gene expression using qPCR. Reprinted with permission under the terms of the Creative Commons CC BY license from reference 52. Copyright © 2023, The Author(s), published by American Association for Cancer Research, Philadelphia, USA.

Their research has also suggested that UA can disrupt KRAS-dependent PI3K/AKT/mTOR signaling, reducing stromal fibrosis and enhancing adaptive T-cell responses 52 **(Figure 8).** UA was confirmed to reduce the tumor burden in a preclinical PDAC mouse model by attenuating M2-like tumor-associated macrophages and increasing the number of memory-like CD4+ and CD8+ T cells. Furthermore, combining UA with anti-PD-1 therapy enhances antitumor responses, promotes Th1 cell expansion, and improves the survival of mice. Interestingly, combination therapy with UA and gemcitabine improved survival outcomes compared with gemcitabine alone, suggesting its therapeutic potential in PDAC management.

### 2.6. Prostate cancer

Prostate cancer ranks among the most frequently diagnosed malignancies in men. It poses a substantial clinical challenge owing to its biological heterogeneity and the development of resistance to standard therapeutic interventions. Initial clinical investigations have provided evidence for the chemopreventive properties of UA 53-54. A phase II clinical trial demonstrated that UA derived from pomegranate juice slowed the prostate-specific antigen (PSA) doubling time in patients with prostate cancer. Notably, UA accumulates in prostate tissues and is correlated with reduced 8-hydroxy-2′-deoxyguanosine (8-OHdG) levels, suggesting that it can modulate oxidative DNA damage 55. Other phase II and phase III trials evaluated the chemopreventive effects of pomegranate extract (its metabolite being UA) in men with favorable-risk prostate cancer via active surveillance 56-57. In a 12-month trial, UA levels were significantly elevated in plasma and urine, and prostate tissue analysis revealed reductions in oxidative DNA damage and androgen receptor expression 56. No significant adverse effects were observed in this study, highlighting the safety and efficacy of UA 57.

Previous studies have shown that UA (40 µM) exerts its anticancer effects by modulating the cell cycle and inducing apoptosis. In LNCaP prostate cancer cells, UA treatment increased the number of cells in the G1 phase of the cell cycle, induced apoptosis, and activated caspases 3 and 7 58. A human genome array confirmed the upregulation of genes such as CDKN1A and FN-1, which are involved in cell cycle regulation and apoptosis. Similarly, in PC-3 and DU-145 prostate cancer cells, UA inhibited cell growth by arresting cells in the G2/M phase through the activation of the Cyclin B1/EDC2 complex via phosphorylation 59. Eska et al. 60 also reported that the anticancer activity of UA is based on cell cycle regulation.

A study by Seeram et al. 61 confirmed the role of UA in reducing the proliferation of prostate cancer cells and inhibiting LAPC-4 prostate cancer xenograft growth. UA preferentially accumulates in prostate and intestinal tissues, suggesting its targeted bioactivity. UA was shown to be an inhibitor of CYP1B1, and prolonged exposure to UA increased its inhibitory effects on 22Rv1 prostate cancer cells 62. The cytochrome P450 enzyme CYP1B1 plays a critical role in prostate cancer development. Notably, urolithins demonstrated cytotoxic effects at concentrations near their CYP1B1 inhibitory IC50 values. Another in vivo study revealed UA activity as a therapeutic agent via the targeting of miR-21 63. At an 80 mg/kg dose, UA administration resulted in a greater than 60% reduction in tumor growth in a DU-145 xenograft mouse model. UA induces caspase-dependent apoptosis and mitochondrial depolarization and reduces the BCL2/BAX ratio. It also downregulates miR-21 expression, leading to the upregulation of the PTEN and Pdcd4 proteins, the suppression of Akt phosphorylation, and the activation of FOXO3a. Additionally, it can inhibit Wnt/β-catenin signaling by reducing MMP-7 and c-MYC expressions, effects that are attenuated by miR-21 overexpression.

Androgen receptor (AR) signaling is central to prostate cancer progression. UA selectively inhibits AR-positive prostate cancer cells by suppressing AR signaling, and its efficacy has been confirmed in enzalutamide-resistant cells 64. This selective effect was verified in vivo in AR-positive xenografts, highlighting AR as a primary target. Furthermore, immunohistochemical analysis further confirmed the downregulation of AR/pAKT signaling in UA-treated tumors. UA has been shown to induce additive antiproliferative activity when combined with androgen inhibitors 65. UA can induce anticancer activities upon modification to its newer analogs 66. For example, ASR-600, a UA analog, effectively inhibited AR and AR-V7 signaling in prostate cancer cells 67. Owing to its selectivity, ASR-600 binds to the N-terminal domain of AR for ubiquitination and degradation, as confirmed by biomolecular and subcellular studies. Compared with C4-2B (AR-V7-resistant, ~650 mm3 vs control, ~1150 mm3) xenograft models, the ASR-600 is also orally available and significantly suppresses tumors in 22Rv1 cells (~100 mm^3^ vs control, ~800 mm^3^).

The tumor suppressor protein (p53) is often inactivated in prostate cancer due to MDM2 overexpression. UA exhibited anticancer activity by restoring p53 function through the inhibition of the p53-MDM2 interaction and p53 polyubiquitination. In both p53+/+ and p53-/+ prostate cancer cell lines, UA induced apoptosis and upregulated the levels of p53 and its targets, including p21, PUMA, and NOXA. In p53-null cells, UA acts via p53-independent mechanisms, modulating the expression of p21, p14ARF, MDM2, and XIAP. These findings underscore the potential of UA in targeting diverse prostate cancer subtypes.

### 2.7. Other cancers

In addition to its effects on breast, colon, glioblastoma, liver, pancreatic and prostate cancer, the potent, multitarget chemopreventive actions of UA can be extended to the management of various other cancer types.

Similar to its effects in various other cancer cell lines, UA exhibits potent anticancer activity in human bladder cancer T24 cells, with a recorded IC50 of 43.9 μM 68. This compound reduces oxidative stress by lowering the levels of intracellular reactive oxygen species and malondialdehyde (lipid peroxidase product) while simultaneously increasing superoxide dismutase activity. Moreover, UA effectively modulated key signaling pathways by decreasing MEKK1 and phosphorylated c-Jun expression while increasing phospho-p38 MAPK and PPAR-γ protein levels. Ultrastructural evaluation of UA-treated cells via transmission electron microscopy confirmed the induction of apoptosis, revealing its mechanistic role as an anticancer agent in bladder cancer.

UA efficiently regulates pigmentation in B16 melanoma cells by reducing melanin production to 55.1% of control levels at a non-cytotoxic concentration 69. This action effectively inhibits the proliferation of melanoma cells. UA demonstrated comparable efficacy to kojic acid, a known skin-whitening agent. These effects were achieved through direct enzymatic suppression rather than changes in tyrosinase gene expression (the IC50 value for tyrosinase inhibition by UA was 19.2 μM). These events indicate that UA is a potential natural tyrosinase inhibitor for depigmentation therapies.

Like in many other studies, UA also exhibits promising activity against non-small cell lung cancer (NSCLC). In studies involving A549, H1299, and H1975 lung cancer cell lines, UA treatment (0.1-100 μM) dose-dependently inhibited cell proliferation 70. Proteomic analysis of A549 cells revealed that UA significantly altered the expression of 128 proteins, with 96 downregulated and 32 upregulated. Pathway enrichment analysis revealed that oxidative phosphorylation, cAMP signaling, and actin cytoskeleton regulation were the key pathways affected. This method revealed that UA treatment reduced the expression of TMSB10, a known cancer biomarker in NSCLC. In addition, UA treatment reduced tumor growth from ~ 400 mm^3^ to ~ 200 mm^3^, and these effects were due to downregulation of TMSB10 protein levels. Another study by Li et al. 70 revealed that UA efficiently inhibits EMT in lung cancer by targeting Snail (a key regulator of EMT), thereby reducing cancer cell migration and invasion. Functional, western blot, and q‒PCR analyses further confirmed these effects. Mechanistically, UA disrupts the p53-MDM2 interaction, leading to Snail ubiquitination and subsequent degradation, providing a novel mechanism for its anticancer efficacy in lung cancer.

UA has potent antiproliferative effects and inhibits the invasion and migratory activity of human HuCCT-1 and SSP-25 cholangiocarcinoma cell lines. Autophagy modulation was identified as a major contributor to UA activity. This finding was verified by the significant downregulation of AKT/WNK1 expression, increased LC3-II expression, and increased autophagosome formation. This enhanced therapeutic benefit was confirmed in a HuCCT human xenograft cell line mouse model, in which treatment with UA (20 mg/kg, 3 times a week) restricted tumor growth to under 125 mm^3^ compared with 450 mm^3^ in control tumors.

A structure-guided merging approach led to the development of an analog of UA, 4-bromo-3,8-dihydroxybenzo[c]chromen-6-one 71. This analog was confirmed to be a novel CK2 inhibitor (IC50, 0.31 µM). The activity of this molecule has been tested in human anaplastic large-cell lymphoma cell lines. A specific scaffold of UA was also verified for various protein kinases, such as nCK2, PIM1, HIPK2, PKA, AURORA, DIRK 1a, GCK, nCK1, and GSK3β.

## 3. Sensitization to treatments

UA has emerged as a potent chemosensitizing agent that enhances the efficacy of conventional cancer therapies. Its ability to interfere with drug resistance mechanisms, modulate immune responses, and protect against treatment-induced toxicity underscores its versatility as an adjuvant in cancer therapy. UA has been shown to increase the therapeutic potential of chemotherapeutic agents such as paclitaxel, 5-fluorouracil (5-FU), cisplatin, and mitoxantrone.

In KYSE30 esophageal cancer cells, UA increased the efficacy of chemotherapies (paclitaxel and cisplatin), ionizing radiation, and hyperthermia 72. This approach represents a feasible strategy for the use of UA as an adjuvant to counteract the adverse effects of conventional treatments. UA has been characterized as a substrate for BCRP/ABCG2 30. This property interferes with ABCG2-mediated drug efflux, potentially enhancing the intracellular retention of mitoxantrone and thus achieving superior therapeutic efficacy in drug-resistant breast cancer cells.

**Figure 9:**
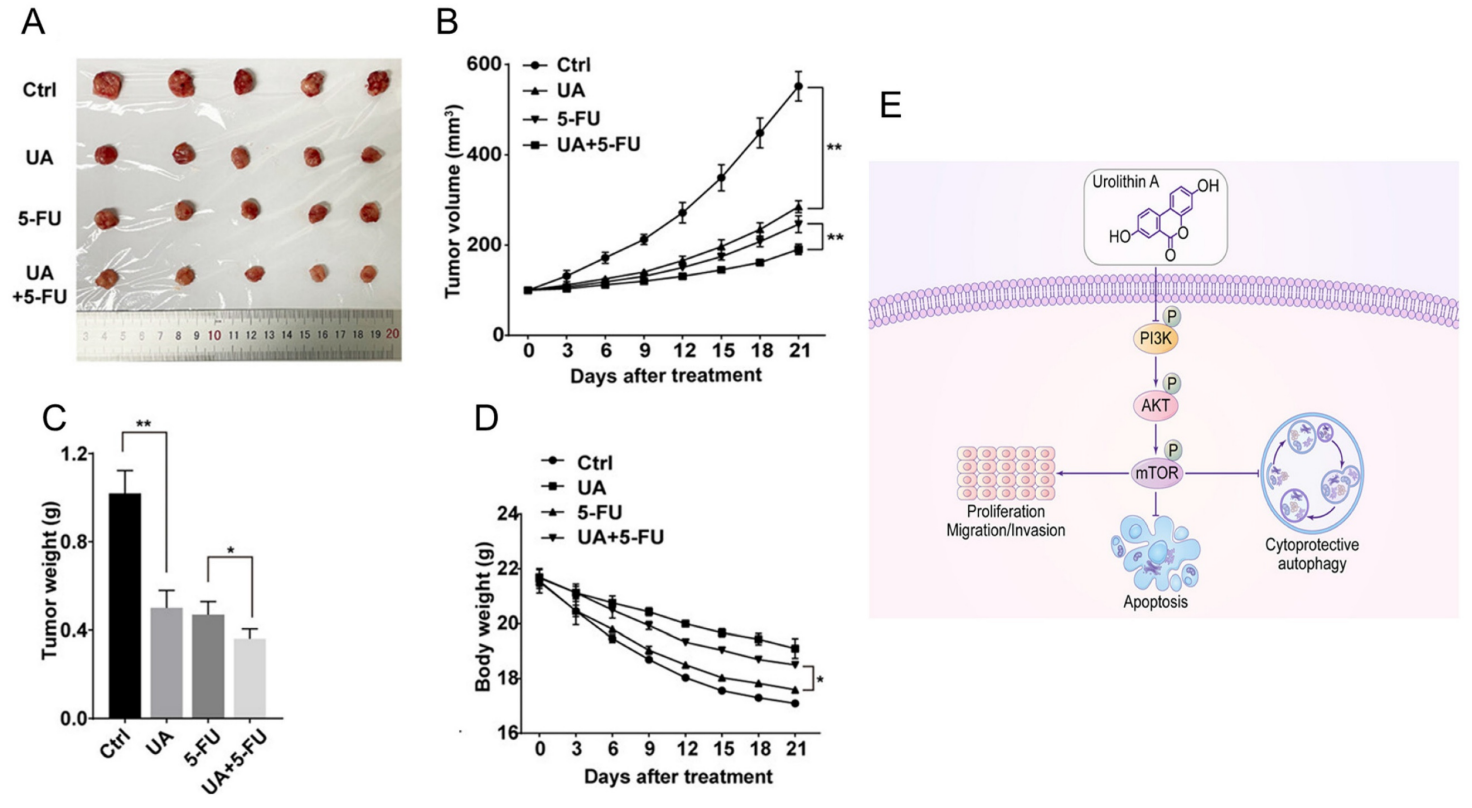
Urolithin A inhibits tumor growth and modulates PI3K/AKT/mTOR signaling in gastric cancer.** A)** Tumors excised and photographed after 21 days of UA treatment. **B,D)** Tumor volumes and body weights of treated mice were monitored every 3 days. **C)** Graphical presentation of tumor weights of different groups treated mice. **E)** Chart of diagram shows antitumor mechanism of UA and its modulation of the PI3K/AKT/mTOR signaling pathway. Reprinted with permission from reference 75. Copyright © 2022, The Author(s), published by John Wiley and Sons.

While 5-FU is a standard treatment for colon and other cancers, its effectiveness is often limited in advanced-stage disease. A previous study confirmed the effects of UA on cancer cell viability and its potential role in enhancing chemotherapy 49. UA moderately inhibited cell viability in breast cancer lines and had selective, mild effects on pancreatic cancer cell lines. In combination studies, UA exhibited slight synergy with 5-FU in KM12 cells, despite the absence of MET induction. These findings reveal that the effects of UA are cell line-specific, and that MET induction does not consistently correlate with increased chemosensitivity, warranting further investigation. UA sensitizes human Caco-2, SW-480 and HT-29 colon cancer cells to 5-FU 73. UA not only reduced the IC50 values of 5-FU but also efficiently arrested the cell cycle at the G2/M phase and amplified apoptosis through increased activation of caspase 8 and caspase 9. Ghosh et al. 43 outlined promising chemosensitizing effects of UA in overcoming 5-FU in SW480 and HCT-116 cell lines and preclinical animal models. Compared with 5-FU monotherapy, the UA+5-FU combination significantly suppressed cell proliferation and invasion and enhanced apoptosis. This profound chemosensitization effect was achieved through the downregulation of drug transporters (MDR1, BCRP, MRP2, and MRP7), the reduction of 5FU efflux, and the modulation of the FOXO3-FOXM1 axis. The involvement of UA and its novel analog UAS03 also significantly reduced the growth of 5FU-resistant cancer cell lines 74. The combination index was less than 1, suggesting that the combinatory action is synergistic in nature. These events occurred due to a reduction in drug transporters and the modulation of EMT-associated markers. In vivo studies further validated the synergistic potential of UA and 5-FU 75 **(Figure 9A-D)** via PI3K/AKT/mTOR signaling **(Figure 9E).** In MKN-45 xenograft mouse models, the combination of UA (20 mg/kg/day via oral gavage) and 5-FU (30 mg/kg twice weekly intraperitoneally) resulted in significantly greater tumor growth inhibition than either agent alone, with tumor volumes decreasing to approximately 180 mm³ in the combination group versus 550 mm³ in the control group. The order of tumor growth was UA+5-FU (~180 mm^3^) > 5-FU (~220 mm^3^) > UA (~280 mm^3^) > control (~550 mm^3^) **(Figure 9 A-C).** In addition, UA+5-FU treatment did not influence on the body weight of mice **(Figure 9D)**.

In pancreatic cancer, UA shows promising antitumor and chemosensitizing characteristics 51 by disrupting the immunosuppressive TME. UA reduces the numbers of tumor-associated macrophages, regulatory T cells, and myeloid-derived suppressor cells, thereby alleviating immune suppression. Preclinical PKT mouse models demonstrated that UA significantly reduces tumor growth, enhances survival, and is well tolerated at physiologically relevant doses (UA 20 mg/kg daily and gemcitabine 20 mg/kg per 3 days). Unlike gemcitabine, UA effectively modulates immune cell infiltration and offers superior therapeutic benefits as a monotherapy, warranting further investigation as a clinical treatment for PDAC. A recent finding suggested that UA is a potential adjuvant to enhance immune checkpoint blockade efficacy in patients with PDAC 52. In a genetically engineered mouse model, Uro A decreased the number of immunosuppressive M2-like macrophages and increased the infiltration of memory-like CD4+ and CD8+ T cells while reducing PD-1 expression. Combining Uro A with anti-PD-1 immunotherapy further increased the Th1 cell infiltration and significantly improved survival.

In LNCaP prostate cancer cells, UA effectively works with the antiandrogen agent bicalutamide 65. This additive effect involves reducing DHT-induced PSA secretion and promoting cytoplasmic localization of the androgen receptor. UA and 5-(3',4',5'-trihydroxyphenyl)-γ-valerolactone (M4) induce modest antiproliferative effects, but their combination has synergistic effects 66. The reason for such actions is enhanced PSA suppression, increased AR cytoplasmic retention, and increased Akt phosphorylation at Ser473. Another study reported that elevating a lower concentration of UA did not yield a meaningful synergistic effect with docetaxel or cabazitaxel in prostate cancer cells 76.

UA enhances immunomodulation to strengthen anticancer immunity 51-52, 77. This action is primarily based on targeting FOXO1 in CD8+ T cells, which drives the nuclear localization and transcriptional activity of FOXO1, increasing CD62L expression and expanding the naïve T-cell population. UA can also mitigate FOXO1 phosphorylation to achieve a sustained functional role in immune defense. Similarly, such antitumor immunity was also achieved by hijacking the ERK1/2-ULK1 cascade to enhance CD8+ T-cell functions via UA 78. Another validation study revealed the significant impact of UA in activating immune cells 79.

UA not only acts as a chemosensitizer but also acts as a protective agent against drug-induced and ionization-induced toxicity 9, 80-83. For example, UA has neuroprotective effects on cisplatin-induced nephrotoxicity by targeting inflammatory and apoptotic pathways involved in kidney injury 80. It significantly suppresses tubular apoptosis and reduces the expression of markers of immune activation, such as T cells, Ig, mucin domain-containing protein-1, and ionized calcium-binding adapter molecule 1. UA also restores endothelial nitric oxide synthase activity and attenuates NF-κB-mediated inflammation, leading to improved kidney function and morphology. UA is also capable of mitigating cisplatin-induced nephrotoxicity by reducing renal oxidative/nitrative stress 81. This was confirmed by a decrease in lipid peroxidation and protein nitration, along with a decrease in proinflammatory cytokines such as TNFα, IL-23, and IL-18. UA also limits CD11b-positive macrophage infiltration in the kidneys and preserves proximal tubular cell integrity. UA has been utilized as a protective agent to mitigate doxorubicin-induced liver and cardiovascular injuries 9, 82.

**Figure 10:**
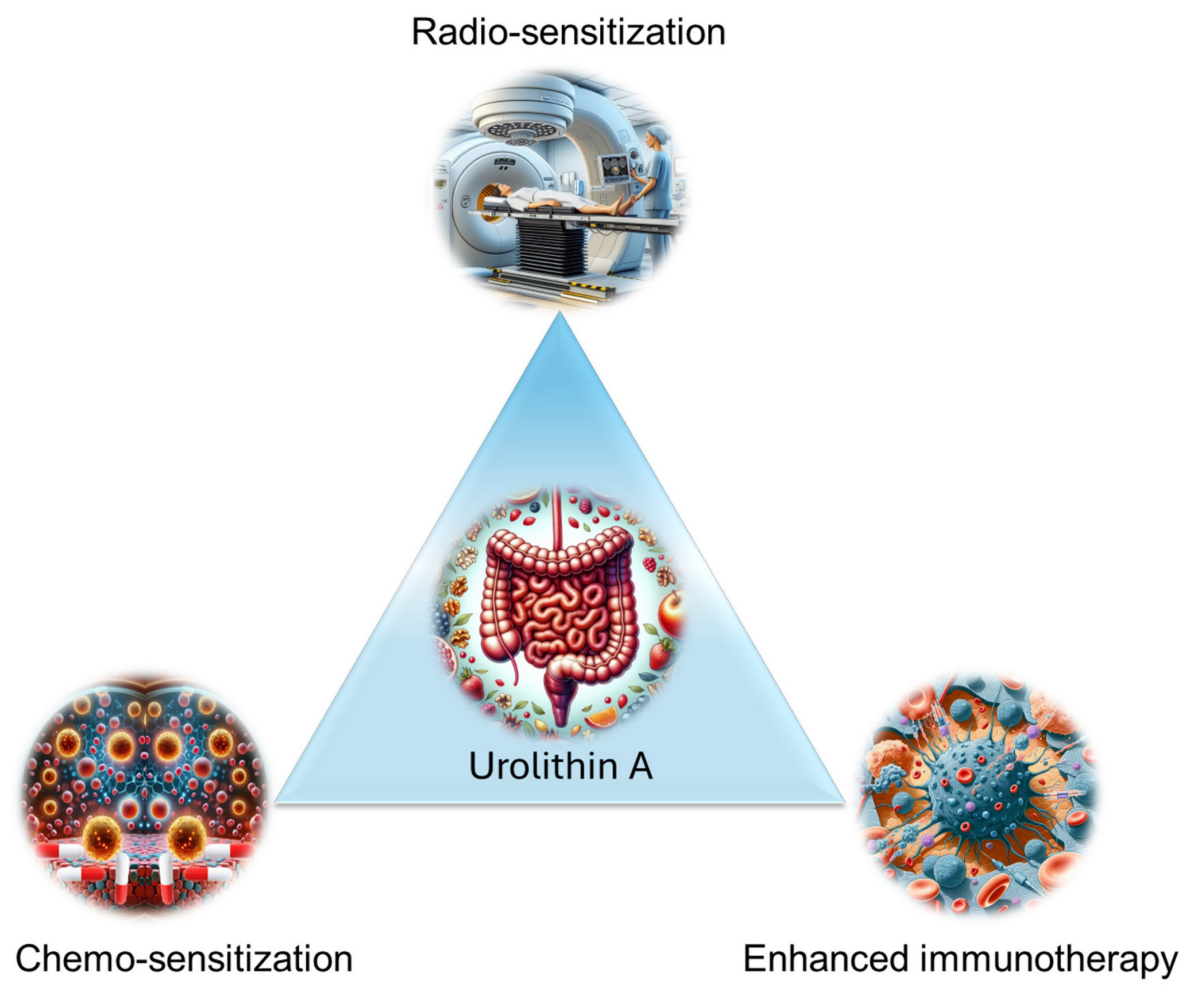
Schematic illustration of the role of UA in chemotherapy, radiation, and immunotherapy enhancement.

Together, UA is a promising therapeutic adjuvant that enhances the efficacy of standard cancer treatments (chemotherapy, radiation, and immunotherapy) **(Figure 10).** Additionally, it offers protection against treatment-associated toxicity. Its ability to modulate immune responses, interfere with drug resistance, and synergize with chemotherapeutic agents underscores its potential for clinical translation. These findings warrant further investigation to optimize its use in combination with other cancer therapies.

## 4. Nanotechnology

UA is poorly soluble in water but readily soluble in organic solvents like ethanol, dimethyl sulphoxide, dimethyl formamide up to 30 mg/ml. However, its solubility can be achieved with carboxy methyl cellulose or polyethylene glycol based aqueous solutions. UA, similar to its parent compound ellagic acid, exhibits poor absorption following oral administration and undergoes rapid elimination due to extensive first-pass metabolism and limited enterohepatic recirculation 84-86. These processes contribute to a reduced plasma half-life and limited tissue bioavailability 87-88. Therefore, UA is expected to exhibit similar pharmacokinetic properties. A human clinical trial reported that only 12% of the studied population exhibited detectable levels of UA glucuronide 88. Furthermore, approximately 40% of participants displayed UA glucuronide plasma levels exceeding 100 ng/mL, while the remaining 60% were either unable to convert UA (33%, with undetectable levels) or were classified as poor converters (27%, with plasma levels <100 ng/mL). These findings underscore the necessity of efficient delivery strategies or supplementation to achieve meaningful preventive or therapeutic benefits of UA.

**Figure 11:**
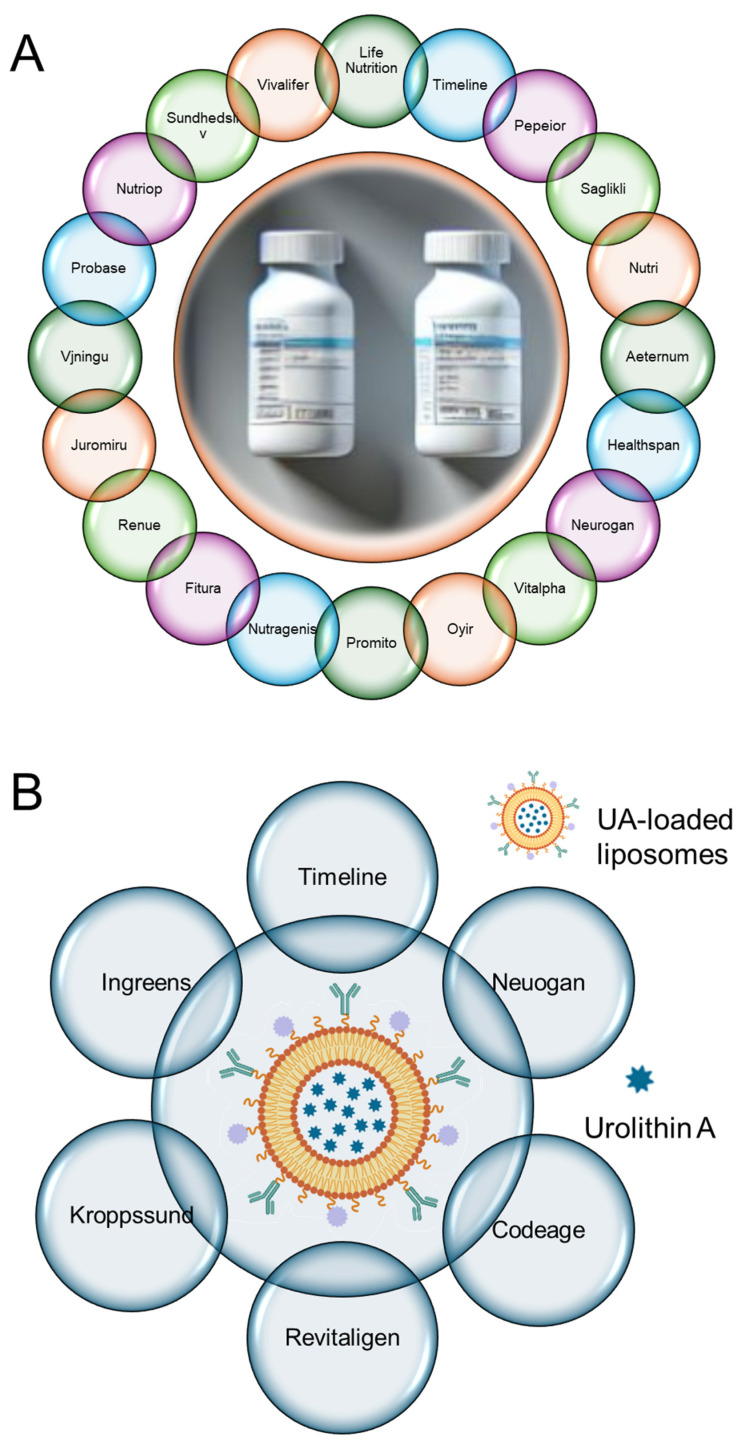
The landscape of UA and its formulations are available on the market as supplements for various symptoms. **A.** Pictorial representation of pill, tablet, and solution formulations of UA in the center surrounded by the selling company names. **B.** UA encapsulated in a liposome in the center surrounded by selling company names.

Although UA has low toxicity in normal cells and substantial antineoplastic potential, its clinical application faces challenges, particularly in terms of bioavailability and tumor targeted delivery. Developing strategies to enhance UA delivery is crucial for improving its bioavailability, either in its natural form or using synthetic and semisynthetic derivatives. This can be achieved by leveraging nanotechnological approaches utilizing innovative carrier systems. To enhance the efficient delivery of UA, it is essential to employ advanced formulation strategies, including UA derivatives, cyclodextrin complexation, nanoparticles, liposomes, niosomes, and polymeric dispersions, similar to those utilized for its parent compound, ellagic acid 89. Currently, UA-based supplements exist in the form of pills, tablets, and solutions** (Figure 11A)**, while some products are also available in the form of liposomal formulations **(Figure 11B)**.

Nanotechnology has revolutionized drug delivery systems, enabling the encapsulation of therapeutic agents within nanoscale carriers to improve their stability, solubility, and bioavailability. Key nanomaterials, such as poly(D,L-lactide-co-glycolide), poly(vinyl pyrrolidone), chitosan, cellulose, poly(ε-caprolactone), and polymer micelles, serve as versatile matrices to control drug release, enhance biocompatibility, and protect therapeutic agents from enzymatic degradation. Solubility enhancers and surfactants, including cyclodextrins, polyvinyl alcohol, dodecyl dimethyl ammonium bromide, and Tween-80, play critical roles as stabilizers during nanoparticle synthesis, mitigating aggregation and improving encapsulation efficiency. These strategies collectively ensure sustained and targeted therapeutic delivery, maximizing clinical outcomes while minimizing off-target effects.

Cyclodextrins have emerged as promising excipients in the production of urolithin complexes, enhancing their solubility, stability, and bioavailability 90. An international publication also aimed at utilizing a dry solid composition of various solubilizing and stabilizing agents, including cyclodextrins 91. Another patented invention describes a method for producing urolithin derivatives by selectively removing the hydroxyl group, resulting in a distinct urolithin compound 92. Such biotransformation is mediated by microorganisms capable of converting the first urolithin to the second in a solution containing the precursor. Cyclodextrins enhance this process by forming inclusion complexes with urolithins, improving solubility and stability, and protecting them from degradation during microbial transformation. This approach offers a novel strategy for producing bioactive urolithin derivatives with improved therapeutic and pharmaceutical properties.

A polymer-based nanoparticle system was generated, and concurrent encapsulation of UA was achieved via an oil-in-water emulsion method 93. For this method, a series of polylactide-polyethylene glycols of different molecular weights in the presence of two different crosslinkers (promellitic dianhydride and cyclohexanetetracarboxylic dianhydride) were utilized 94. This method significantly increased the oral bioavailability of UA, which doubled when it was conjugated with gambogic acid 93.

Liposomal drug carriers have been extensively validated for the delivery of anticancer agents, including doxorubicin, with reduced systemic toxicity and improved targeting. FDA-approved liposomal formulations, such as Doxil® and Daunoxome®, exemplify their clinical relevance. A PEGylated liposomal formulation of UA was developed using 1,2-distearoyl-sn-glycero-3-phosphoethanolamine-conjugated polyethylene glycol 2000, soy phosphatidylcholine, and cholesterol 95. The formulation achieved a particle size of ~122 nm, a negative zeta potential, and an encapsulation efficiency exceeding 94%. These UA-loaded liposomes demonstrated enhanced cellular uptake, cytotoxicity, and apoptosis in HepG2, Huh-7, and Hep3B liver cancer cells. Pharmacokinetic studies revealed improved bioavailability, with UA-PEG-liposomes exhibiting superior plasma exposure compared with free UA and non-PEGylated liposomes. Compared with that of UA alone, pharmacokinetic evaluation revealed a prominent increase in drug exposure to the plasma of rats when a single dose (10 mg/kg) of UA-liposomes or UA-PEG-liposomes was administered. The area under the curve (h*µg/ml), half-life (h), and mean residential time (h) parameters were as follows: UA-PEG-lipids (241, 6.78, 5.88) > UA-lipids (142, 2.56, 3.79) > UA (96, 1.48, 2.42). Another study reported that pH-driven methods enhanced UA encapsulation 96. This approach offered improved storage, thermal, pH, and gastric intestinal digestion stability while enhancing the pharmacokinetics of UA. The formulations are milky, and the morphology of the encapsulated particles resembles cubes under a transmission electron microscope.

Considering this concept, this team also explored the use of prebiotic saccharides (gum arabic, fructooligosaccharide, konjac glucomannan, and inulin)-coated liposomal formulations to enhance the stability and bioaccessibility of UA 97. Encapsulation in liposomes improved UA's encapsulation efficiency (EE) and physicochemical stability, with gum arabic-coated liposomes showing the highest EE and bioaccessibility. Fructooligosaccharide-coated liposomes exhibited superior freeze-thaw stability. Additionally, prebiotic saccharides modulate gut microbiota by promoting beneficial bacteria while reducing harmful ones. These findings suggest that prebiotic-based UA formulations could improve drug stability and support cancer prevention and therapy.

Another attempt was made to construct UA-based solid lipid nanocarriers 25. The uniqueness of this formulation is that lipids are decorated with folate-linked chitosan as layers. This approach promotes cancer cell-specific binding and internalization, which has been confirmed with breast (MCF-7 and MDA-MB-231), liver (HepG2), and pancreatic (PANC-1) cancer cell lines to induce effective UA actions. However, there was a negligible effect on the AGS and HDF normal cell lines. This novel formulation efficiently inhibits ABTS free radicals and DPPH free radicals while increasing catalase gene expression. This solid lipid nanoformulation of UA also promoted the apoptotic potential of cancer cells. Paula et al. 98 aimed to engineer UA-loaded polymer- and lipid-based hybrid nanoparticles to produce the best formulation. For this comparative study, P-NP (PLGA-GA) emulsion‒diffusion evaporation (157 nm size and 52% encapsulation of UA), L-NP (TPGS and tristearin) solvent evaporation (132 nm size and 70% encapsulation of UA), and H-NP (PLGA-GA with tristearin) emulsion‒diffusion epoxide (186 nm size and 29% encapsulation of UA) techniques were implemented. This study also includes an evaluation of the stability and cellular uptake benefits of such advanced encapsulation approaches.

A feasibility study reported a cerium oxide nanoparticle for the conjugation of urolithin via an amine group 99. A systematic characterization by particle size, zeta potential, and spectral-based confirmations was conducted. Additionally, upon conjugation, the cytotoxicity of urolithin remains preserved in multiple functional assays, such as proliferation, the cell cycle, apoptosis, and ROS generation. Excipient-free and spray-drying technology was implemented to obtain UA microparticles 100. These particles were optimized to obtain useful physicochemical properties. Initial in vitro studies demonstrated the biocompatibility, safety, and ability of UA to maintain epithelial barrier integrity across pulmonary cell models, which are highly useful for localized lung cancer applications.

Although directed studies on "urolithin A" and nanoparticles are limited, extensive research on "ellagic acid" and “nanoparticles” 101 (87 articles resulted in a PubMed search, data acquired on December 17, 2024) and “ellagitannins” and “nanoparticles” (9 articles resulted in a PubMed search, data acquired on December 17, 2024) 102 provides valuable insights. Nanocarriers such as titanium/zinc oxide, silver, copper, gold, magnetic nanoparticles, protein shells, chitosan, lipid nanocarriers, and in situ gelling nanosystems have been successfully employed for these compounds. Emerging delivery platforms, such as gambogic acid- and folate-conjugated nanoparticles, further increase the cytotoxic specificity and delivery efficiency of UA to tumors. These advances position UA-based nanotechnology as a promising approach for addressing its bioavailability limitations and advancing its therapeutic applications. Future research should focus on expanding UA-loaded nanocarrier systems, leveraging innovative methodologies, and conducting robust preclinical and clinical studies to transition these technologies from bench to bedside.

## 5. Conclusions and future perspectives

Urolithin A, a metabolite derived from ellagitannins, has emerged as a multifaceted therapeutic agent with robust potential in cancer prevention and therapy. By targeting hallmarks of cancer such as oxidative stress, inflammation, cellular senescence, and dysregulated signaling pathways, UA offers a unique mechanism of action that spans multiple cancer types, including breast, colorectal, and pancreatic cancers. Its ability to modulate critical oncogenic pathways, enhance mitochondrial function, and induce mitophagy underscores its therapeutic value. Despite this promise, challenges such as low bioavailability and rapid gastrointestinal degradation remain critical barriers to clinical translation. Future directions should focus on rationally designing UA derivatives with improved potency, stability, and selectivity. Chemical modifications, such as methylation, glycosylation, or ligand conjugation, could optimize its pharmacokinetics and enhance tumor targeting. Additionally, scalable production techniques and integration with established nanocarrier systems are essential for clinical readiness. Exploring UA's role in combination therapies and its potential to modulate the tumor microenvironment will further expand its applications in precision oncology.

The integration of UA into nanotechnological platforms offers a promising approach to overcome its limited bioavailability, rapid metabolism, and poor tissue distribution. Nanocarriers, polymeric dispersions, liposomes, and nanoparticles enable targeted delivery, controlled release, and enhanced tumor accumulation, optimizing UA's therapeutic efficacy. These nanoformulations can improve its gastrointestinal stability and absorption. Developing tabletized nanomedicines for UA encapsulation offers additional benefits, such as ease of administration, scalable manufacturing, and extended shelf life. Tailored delivery platforms, such as tumor microenvironment-responsive or pH-sensitive carriers, could further refine UA's therapeutic precision while minimizing systemic side effects. Drawing inspiration from FDA-approved nanomedicine platforms like Abraxane and Doxil, UA or its analogs-based nanoformulations can leverage established safety profiles to streamline regulatory approval and clinical adoption. The integration of UA into multifunctional nanomedicine systems represents a transformative step in cancer therapy. Combining UA with imaging or theranostic functionalities could facilitate real-time tumor monitoring, enabling a dual therapeutic-diagnostic approach. Furthermore, UA's potential synergy with chemotherapy, radiation, and immunotherapy offers a promising avenue to enhance efficacy, overcome resistance, and reduce adverse effects.

Combining UA with cell cycle inhibitors, antibody therapies, and PD-L1 inhibitors in nanoformulations can further potentiate its anticancer effects. Cell cycle inhibitors disrupt tumor proliferation, while antibody therapies enhance tumor specificity. UA combined with PD-L1 inhibitors may enhance immune responses, improving the efficacy of immunotherapy. These multi-modal strategies address cancer resistance and immune evasion mechanisms. Additionally, enzyme-responsive nanocarriers designed to respond to TME stimuli, such as pH, enzymes, and redox gradients, offer precise drug localization, overcoming conventional treatment limitations. Key challenges remain in enzyme variability, tumor boundary identification, nanocarrier scalability, and protein corona formation during intravenous administration. Future research should focus on refining nanocarrier designs, optimizing surface modifications, and conducting rigorous preclinical and clinical evaluations. Combining UA with targeted therapies can provide a synergistic approach to revolutionize cancer treatment and improve patient outcomes.

In conclusion, UA embodies a paradigm shift in natural product-based cancer therapies, offering a comprehensive approach that combines mitochondrial health, immune modulation, and synergistic therapeutic potential. Addressing bioavailability challenges through advanced nanotechnology and validating its safety and efficacy in clinical trials will be critical to establishing UA as a cornerstone of personalized and precision cancer medicine. This trajectory positions UA as a promising candidate for future research and clinical innovation, with the potential to revolutionize the landscape of oncology.

## Figures and Tables

**Table 1:** Summary of peer-reviewed review articles investigating the diverse biological functions and therapeutic roles of urolithin A.

Title of review article, Primary Author, and reference	Areas covered in the review article, its significance, and overall conclusions
Antioxidant and Anti-Inflammatory Properties of Walnut Constituents: Focus on Personalized Cancer Prevention and the Microbiome Fan et al., 2023 ^12^	This article critically evaluates the impact of ellagitannins and their gut microbiota-derived metabolites, such as Urolithin A, on cancer risk and inflammation. It explores how these compounds modulate cellular pathways that promote anticancer and antioxidant activities, highlighting their potential role in mitigating oxidative stress and inflammation as part of a broader strategy to reduce cancer risk.
Ameliorative Effects of Gut Microbial Metabolite Urolithin A on Pancreatic Diseases Li et al., 2022 ^13^	This review delves into the metabolism of UA and its biological effects in treating pancreatic diseases. The review discusses how UA activates autophagy, reduces inflammation, and maintains mitochondrial function, as well as its role in modulating the gut-pancreas axis, offering insights into its unique mechanisms for managing pancreatic disorders.
The Therapeutic Potential of Urolithin A for Cancer Treatment and Prevention Vladimir S. Rogovskii, 2022 ^14^	This article presents a detailed examination of the anti-inflammatory and anticancer properties of UA. It discusses UA's potential to act through aryl hydrocarbon receptor antagonism, modulation of protein phosphorylation, and stabilization of p53, positioning it as a promising candidate for both cancer treatment and prevention.
A mechanistic insight into the biological activities of urolithins as gut microbial metabolites of ellagitannins Hasheminezhad et al., 2022 ^15^	The therapeutic potential of Urolithins across a range of diseases, including cancer, diabetes, cardiovascular conditions, and age-related diseases. The article addresses the molecular mechanisms underlying UA's action, such as MDM2-p53 inhibition, MAPK modulation, and NF-κB suppression, alongside their pharmacokinetics, and highlights their emerging significance as pharmacological agents for disease management.
Urolithins: Diet-Derived Bioavailable Metabolites to Tackle Diabetes Raimundo et al., 2021 ^16^	This work investigates UA's role in diabetes management, focusing on the metabolic pathways it influences and its mechanisms of action. This highlights the therapeutic potential of UA in addressing chronic diseases, emphasizing its capacity to improve insulin sensitivity and metabolic health.
Targeting PI3K Pathway in Pancreatic Ductal Adenocarcinoma: Rationale and Progress Mehra et al., 2021 ^17^	This article examines Urolithin A's potential as a therapeutic agent for pancreatic cancer, focusing on its ability to target the PI3K/AKT/mTOR pathway. The review also discusses how Urolithin A may enhance therapy response through immune microenvironment modulation, making it a promising candidate for improving pancreatic cancer treatment outcomes.
Potential of the ellagic acid-derived gut microbiota metabolite - Urolithin A in gastrointestinal protection Kujawska et al., 2020 ^18^	This article documents the potential of Urolithin A in protecting against gastrointestinal (GI) cancer and inflammation. It highlights how Urolithin A modulates tumor pathways, enhances carcinogen biotransformation, and boosts antioxidant defenses, underscoring its therapeutic promise for cancer prevention and treatment, particularly in GI malignancies.
Targeting Autophagy in Aging and Aging-Related Cardiovascular Diseases Ren et al., 2018 ^19^	The role of autophagy in aging, cardiovascular diseases, and cancer, with a particular focus on Urolithin A as an autophagy inducer. It discusses how Urolithin A may improve health span and lifespan while preventing aging-associated diseases, including cancer, by enhancing cellular maintenance and mitigating age-related dysfunctions.
An Overview of Natural Plant Products in the Treatment of Hepatocellular Carcinoma Rawat et al., 2018 ^20^	This article emphasizes the role of dietary phytochemicals, including Urolithin A, in cancer prevention. It discusses the mechanisms by which Urolithin A contributes to cancer prevention, such as scavenging free radicals, inhibiting cell growth, and inducing apoptosis.
Health benefits of walnut polyphenols: An exploration beyond their lipid profile Sánchez-González et al., 2017 ^21^	This article explores the polyphenolic compounds in walnuts, particularly ellagitannins, and their metabolism by gut microbiota into Urolithin A, B, C, and D. The review highlights the antioxidant, anti-inflammatory, and disease-preventive potential of these metabolites, particularly in cancer, cardiovascular, and neurodegenerative diseases, emphasizing the health benefits of walnut-based phytochemicals.
